# Cone Beam CT vs. Fan Beam CT: A Comparison of Image Quality and Dose Delivered Between Two Differing CT Imaging Modalities

**DOI:** 10.7759/cureus.778

**Published:** 2016-09-12

**Authors:** Lawrence Lechuga, Georg A. Weidlich

**Affiliations:** 1 California State University Fresno; 2 Radiation Oncology, National Medical Physics and Dosimetry Comp., Inc

**Keywords:** cbct, fbct, fan beam, image quality, dose, uniformity

## Abstract

A comparison of image quality and dose delivered between two differing computed tomography (CT) imaging modalities—fan beam and cone beam—was performed. A literature review of quantitative analyses for various image quality aspects such as uniformity, signal-to-noise ratio, artifact presence, spatial resolution, modulation transfer function (MTF), and low contrast resolution was generated. With these aspects quantified, cone beam computed tomography (CBCT) shows a superior spatial resolution to that of fan beam, while fan beam shows a greater ability to produce clear and anatomically correct images with better soft tissue differentiation. The results indicate that fan beam CT produces superior images to that of on-board imaging (OBI) cone beam CT systems, while providing a considerably less dose to the patient.

## Introduction and background

### Purpose

The purpose of this paper is to review and compare cone beam CT (CBCT) and fan beam CT (FBCT) and their respective image quality. Better image quality will improve visualization of anatomical detail, increase ability to diagnose disease, and improve accuracy of the image guidance process during radiotherapy. Previous studies by Elstrøm, et al.and Garayoa, et al.^ ^laid the foundation for this work [1-2]. These studies implemented various imaging protocols with both FBCT and CBCT and quantitatively analyzed different aspects of the images. Under clinical pelvis and head and neck protocols, numerous scan modes were implemented. The images were then assessed for various image quality parameters to determine which modalities produced the more desirable images. The images produced by FBCT were shown to be of superior quality in clarity, uniformity, anatomical accuracy, low contrast resolution, and delivery of a lower dose to the patient. The following analysis of the literature leads to the conclusion that FBCT is more desirable for in vivo imaging.

### Background

In 1971, the first prototype computer assisted tomography (CAT) scanner was installed in a hospital in England [3]. This type of scan involved acquiring sequential, thin axial scans through the patient's volume. This method allowed the visualization of soft tissue, which led this technology to become very popular. We will refer to it as FBCT. In 2001, a form of computed tomography became commercially available in the United States and became known as Cone Beam CT (CBCT). CBCT using diverging kV X-rays has the ability to visualize anatomical structures and acquire images over a much larger volume in a single scan than is capable with FBCT. Building on this, a great advancement was made in the field of radiation therapy with the integration of linear accelerator mounted CBCT for radiation therapy units. This integration of CBCT imaging with radiotherapy units has allowed the patient to be imaged directly before therapy. This has the advantage of providing pretreatment verification of patient target and normal tissue anatomy. Therefore, any small changes in the afflicted area’s geometry may be presented prior to delivering the dose. This simultaneously assures that the patient is correctly positioned for treatment. As stated in a study by Verellen, et al., the primary goals of image guided radiotherapy (IGRT) are to optimize the reduction in treatment margins, allow the use of sharp dose gradients that are common with intensity-modulated radiation therapy (IMRT), utilize “dose painting,” and interactively adapt to changes within the lesion at the time of treatment [4]. These goals rely heavily on proper images between the planning CT and verification CBCT and minimal patient movement.

Though CBCT is a very dominant form of IGRT used in radiotherapy, it still exhibits many weaknesses that can affect image quality. As Elstrøm, et al. pointed out, current CBCT acquisition modes are aimed at minimizing dose to the patient [1]. This reduction in dose may prove to be beneficial to the patient, but it may reduce the quality of the images and the accuracy of the assigned Hounsfield unit (HU). Compounding this issue is the intrinsic problem that the large cone geometry produces more artifacts and scatter than the conventional fan beam CT [1]. With this knowledge in mind, it may prove beneficial to objectively compare the image quality and dose delivered by CBCT and FBCT. This comparison can be accomplished by analyzing the quality of reconstructed images, defining specific image quality, and examining absorbed dose statistics that will take subjective bias out of the equation.

## Review

### Methods and materials

Various imaging protocols were designed to investigate differences in image quality of CBCT and FBCT. In the study by Elstrøm, et al., all the CBCT scans were performed with a Varian OBI System (Varian Medical Systems, Inc., CA, USA) on a Trilogy Tx linear accelerator (Varian Medical Systems, Inc., CA, USA) [1]. This system used a kV X-ray source with orthogonal detectors mounted on the gantry. Utilizing a head and neck protocol, the following parameters were used: full-fan cone beam with bow tie filtration, source to detector distance of 1500 mm, 3-mm slice thickness, transversal FOV of 250 mm, and longitudinal FOV of 175 mm. Furthermore, for the fan beam CT, two multi-slice scanners (Mx8000 IDT 16 and BB16), (Phillips Medical Systems, Wisconsin, USA) were used under standard multi-slice CT head and neck protocol. The protocol incorporated the parameters: 250 mm FOV, 512x512 pixel matrix, sharp filter, 3-mm slice thickness, and 16x0.75-mm collimation. Five different scan acquisitions and two different exposures were used for the CBCT and fan beam CT respectively (see Table 1) [1].

**Table 1:** Acquisition Modes Under Head and Neck Protocol Acquisition modes and parameters for CBCT and CT scans under head and neck protocol for the study by Elstrøm, et al. [1]

Image Modality	Acquisition Mode	Peak Voltage (kVp)	Tube Current (mA)	Exposure Time (ms)	Rotation Range (Deg)	Number of Projections	Exposure (mAs)
CBCT	Standard Dose Head (SDH)	100	20	20	200	372	149
	Standard Dose Head Full (SDHFS)	100	20	20	360	669	268
	High Quality Head (HQH)	100	80	25	200	372	744
	High Quality Head Full (HQHFS)	100	80	25	360	669	1338
	OBI1.3 Full Scan (OBI13FS)	125	80	25	360	669	1338
FBCT	Mx8000 IDT (CTMX)	120	-	-	-	-	150/300
	BB16 (CTBB)	120	-	-	-	-	150/300

In a separate study by Garayoa and Castro, images were acquired with both CBCT (Varian OBI system with mounted Varian CLINAC 21EX linear accelerator with orthogonally placed detectors) and FBCT (CT Aquilion LB, Toshiba Medical Systems Corporation, Japan) [2]. Images for CBCT were acquired under the pelvis clinical and pelvis spotlight protocol which used the following parameters respectively: 650/375 projections in 364/200° rotation, detector to source distance of 1500 mm, half-fan/full-fan filter, FOV of 256x256 mm, and a 512x512 reconstruction matrix. Moreover, the images from the fan beam CT were acquired in an attempt to match parameters of the CBCT. The fan beam CT used a helical technique with a pitch of 0.938 and a 512x512 reconstructed pixel matrix with a FOV of 256 mm. The protocol parameters for CBCT and CT can be seen in Table 2 [2].

**Table 2:** Acquisition Modes for CBCT and CT Scans Modes for CBCT and CT scans under pelvis protocols for the study by Garayoa, et al. [2]

Scan Type	Protocol	Peak Voltage	Det. to Source	Current	Pulse width	Rotation (deg)	FOV	Projections
CBCT Half Fan	Pelvis Clinical	125 kVp	150 cm	80 mA	13 ms	364	256x256 mm	650
CBCT Full Fan	Pelvis Spot Light	125 kVp	150 cm	80 mA	25 ms	200	256x256 mm	370
Fan Beam CT	Pelvis Clinical	120 kVp	127.5 cm	80 mA	1 rotation	helical	256x256 mm	-

**Figure 1:**
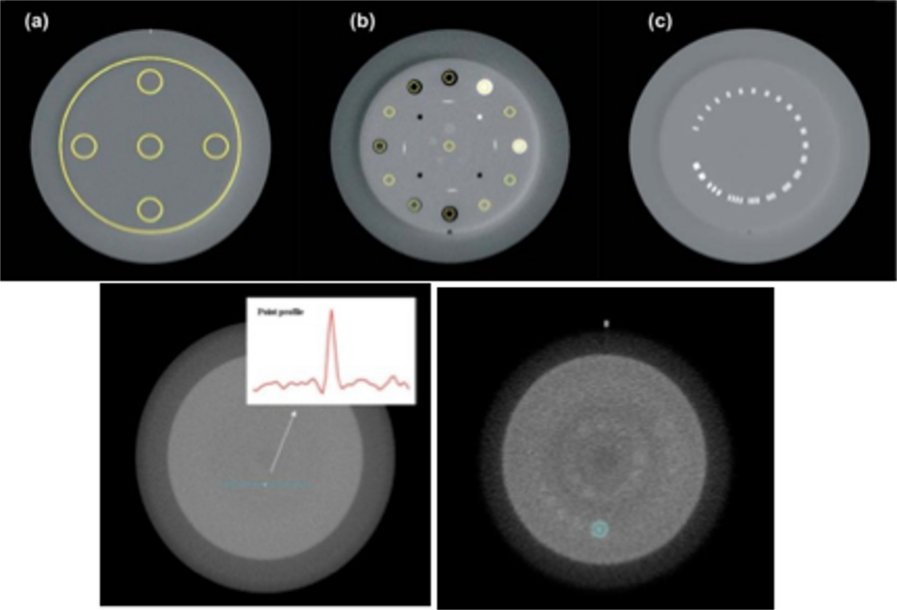
Catphan Modules Used in the Studies Discussed (a) CTP-486 uniform water equivalent, (b) CTP-404 with 12 inhomogeneity inserts, (c) CTP-528 with up to 21 lp/cm [1]. Bottom left: CTP 591 tungsten carbide bead used to analyze spatial resolution. Bottom right: CTP 515 utilizes inserts of various contrasts to evaluate low contrast detectability [2].

In order to evaluate image quality, there have to be some nominal values and features to compare the results to. For this reason the use of a phantom was employed in the studies mentioned earlier. For the study by Elstrøm, et al., the Catphan® 504 phantom (The Phantom Laboratory, NY, USA) was used [1]. This cylindrical phantom used three different modules (see Figure 1) which were the following: CTP 486 uniform water equivalent disk, CTP 404 containing eight inhomogeneity inserts, and the CTP 528 with 21 line pairs/cm [1]. Though similar, Garayoa and Castro’s study used the Catphan® 600 (The Phantom Laboratory, NY, USA), which contains five modules as shown in Figure 1 [2]. These modules were the CTP 404, the CTP 486, the CTP 528, and the CTP 591 with a tungsten-carbide bead embedded into uniform material, and finally the CTP 515 with inserts of varying contrast. Each of these modules have many purposes in evaluating image quality.

It would prove beneficial to discuss each of the phantom modules used in the studies. The CTP 404 uses 12 inserts of different known densities, which can vary from -1000 HU to 990 HU. These inserts can be used to test the linearity of the system and to measure spatial linearity by measuring and comparing the distance between the Teflon and air inserts. The CTP 486 uses uniform material to mimic the density of water [1-2]. Being able to compare the nominal density of the slice to the scan measurement, gives the ability to assess Hounsfield unit uniformity and accuracy. This also gives information on the amount of noise. The CTP 515 module utilizes groups of small inserts of different size and nominal contrast where these contrasts are known to be 1%, 0.5%, and 0.3%. This allows the evaluation of low contrast sensitivity. The CTP 528 incorporates various groups of lines of varying frequencies. These lines are embedded in the slice and vary up to 21 line pairs/cm. This ability of the detector to discriminate the various line frequencies allows for the assessment of spatial resolution. Last but not least, the CTP 591 module uses a bead made of a tungsten-carbide that is embedded in uniform water equivalent material. Similar to CTP 528, this module can also be used to assess spatial resolution [1-2].

As mentioned earlier, evaluating the image quality between CBCT and FBCT may be enlightening. Though there is merit in qualitative comparisons between images, some image quality statistics can be calculated to quantify comparisons. This will allow a quantitative view of the differences between the two imaging modalities. In both the studies by Garayoa, et al. and Elstrøm, et al., all image quality analysis was performed in the ImageJ software (a public domain image-processing program) using the digital imaging and communications in medicine (DICOM) format for the images [1-2]. ImageJ allowed the use of macros to perform various image quality tests. Various aspects of image quality were analyzed such as: spatial resolution, HU uniformity/accuracy, image noise, and low contrast sensitivity. In order to quantify these parameters, various image statistics were developed.

The modulation transfer function (MTF) represents a measure of spatial resolution in the imaging system. This measure is evaluated within the Catphan CTP 528 module [1]. Essentially, using the CTP 528, the system will image the sets of lines of varying frequency. The MTF is a quantitative way to decide how well a device can resolve small spatial changes. Both studies by Garayoa, et al. and Elstrøm, et al., evaluated the MTF, but not in precisely the same way [1-2]. In the study by Elstrøm, et al., they started by calculating the edge spread function (ESF) from 360 different radial profiles [1]. Where the derivative of the ESF gives the line spread function (LSF), which is then Fourier transformed to the modulation transfer function. This MTF is then normalized at '1' for zero spatial frequency and the values at 50% and 10% are estimated. The study by Garayoa, et al. used the same method above but in addition calculated MTF in a second way [2]. Using the CTP 591 module, an image is acquired where the system response to a small tungsten bead is analyzed. The system response to a Dirac-Delta-like object leads to the calculation of the MTF. Further details on the derivation of these quantities like MTF can be found in a study performed by Grimmer, et al [5].

The image uniformity and noise were evaluated in multiple ways using the water-equivalent module. One of the ways Elstrøm, et al. analyzed image uniformity was to compare the mean and standard deviation within the central slice of the CTP 486 module [1]. As mentioned in the previous section, the CTP 486 mimics the density of water, so this gives it a nominal Hounsfield unit value of zero. Therefore, a scan should return a value of zero for each voxel in the image and give a mean value of zero. This method measures the system response and calibration. When the standard deviation from the mean is considered, it will give a quantitative measure of the variation in the detector response as well as the deconvolution process. One other way that uniformity was measured was to define a quantity called the uniformity index (UI). The UI takes the maximum percent difference between the mean HU value of each peripheral region of interest (ROI) and the central ROI respectively (see Figure 1) [1]. The study by Elstrøm, et al. defined UI as the following:

$$UI = 100\cdot \frac{\overline{HU}_{perihpery}-\overline{HU}_{central}}{\overline{HU}_{central}}$$

This image statistic allows the quantification of the amount of “cupping” and “capping” artifact in the image. The study by Garayoa, et al. defined a statistic called the C-value which also quantified the amount of “cupping” and “capping” [2]. A positive value represents capping, while a negative C-value represents cupping.

Another aspect to consider is the signal-to-noise ratio. It is important to not look at solely the level of noise in the images, but to look at the ratio of the “true” signal to the noise signal. The “true” signal is the signal that is representative of the true anatomy of the region of interest. The signal from the noise would represent the amount of signal that is due to random scattering effects. When the signal-to-noise ratio is decreased, that will produce a grainier image. It is generally assumed that the cone-beam imaging modality will intrinsically produce relatively more noise from scattering due to its large cone geometry [1].

Lastly, another aspect of image quality to consider is a system’s ability to detect objects of low visibility or of differing contrast. One way to measure image quality was to define a value known as low contrast visibility (LCV). In the study by Elstrøm, et al. two inserts with the closest HU values, low density polyethylene and polystyrene, were placed in the CTP 404 module to be imaged [1]. From this the LCV was calculated via:

$$LCV = 2\cdot \frac{\overline{HU}(LDPE)-\overline{HU}(PS)}{\sigma(LDPE)+\sigma(PS)}$$

where $\sigma$​ represents the standard deviation in the HU values of the respective inserts [1]. This method allows one to quantify a system’s ability to discriminate small differences in the visibility of materials.

In addition to the feature of image quality, it is worth discussing the dose delivered by the two differing imaging modalities. In a study by Kan, et al. the radiation dose from CBCT and fan beam CT for three different protocols were investigated [6]. This study acquired all CBCT data using a Varian OBI. The scanning protocols used can be viewed in Table 3. Fan beam images were acquired using a GE Lightspeed RT 16 Multislice CT (GE Healthcare, UK). The protocol specifics for the FBCT scans can be found in Table 3. Utilizing a female anthropomorphic RANDO® Phantom (The Phantom Laboratory, NY, USA) and Thermoluminescent dosimeters, or TLD-100 chips (Thermo Fisher Scientific, MA, USA), the absorbed dose for various organs and effective dose to the body was calculated [6].

**Table 3:** CBCT and FBCT Scan Protocols CBCT and FBCT scan protocols used for study by Kan, et al. [6].

	Scan Site	Scan Mode	Field of View	kVp/mAs	Slice Thickness	Longitudinal Extent
CBCT	Head and Neck	Full fan	24 cm	-	2.5 mm	15.5 cm
	Chest	Half fan	40 cm	-	2.5 mm	13.7 cm
	Pelvis	Half fan	40 cm	-	2.5 mm	13.7 cm
FBCT	Head and Neck	Axial 2.5 mm x 4i	25 cm	120/300	-	15.5 cm
	Chest	Axial 2.5 mm x 4i	50 cm	120/265	-	13.7 cm
	Pelvis	Axial 2.5 mm x 4i	50 cm	140/210	-	13.7

To calculate the absorbed dose, the study based the calculation on an Institution of Physics and Engineering in Medicine and Biology (IPEMB) protocol for low-energy X-rays, which resulted in the following [6-7]:

$$D_{w,q}= M_{q}N_{k,q}B_{w,q}[(\tfrac{\mu}{\rho})_{w,air}]_{q}$$

where:

                        q= user’s energy in terms on HVL.

                        N_k,q_ = units of Gy/C, is the air kerma calibration factor for ion chamber at energy q.

                        Mq = corrected electrometer reading in C for the ion chamber at the energy q for a certain treatment cone.

                        B_w,q_ = backscatter factor for water at the surface with energy q.

                       $[(\tfrac{\mu}{\rho})_{w,air}]_{q}$ = mass energy absorption coefficient of water to air at energy q.

The effective dose to the body, which represents the stochastic health effect risks, was calculated with the International Commission on Radiological Protection 60 (ICRP 60) recommendations that follow:

$$D_{eff}= \sum_{T}w_{T} \sum w_{R} D_{T,R}$$

where w_R_ is the weighting factor for radiation type, D_T,R_ is the mean dose absorbed to the organ, and w_T_ is the tissue weighting factor. The doses to various organs were calculated under three protocols: head and neck, chest, and pelvis. Utilizing identical TLD placement, this study was able to compare the delivered dose from CBCT with FBCT [6]. These calculations of dose can help quantify the real possibility of increased risk of tissue damage and secondary cancers from the two differing CT systems.

## Conclusions

### Results

In the comparison performed by Elstrøm, et al. on CBCT vs CT, the edge spread function was used to calculate the MTF for various scans (see Table 1) [1]. As listed in Table 4, it was found that CBCT outperformed fan beam CT in having a greater MTF value at both 50% and 10%. It was suggested by Elstrøm, et al. that a reduction in MTF values on full rotation scans may be a result of increased mechanical instability of the full rotation methods compared to the partial scan methods [1]. These results are consistent with a study performed by McCann, et al. where similar MTF values were found for fan beam CT [8]. However, it is important to note that the MTF values obtained by McCann vary greatly with applied protocol [8]. In addition, the study performed by Garayoa, et al. found similar results comparing CBCT to FBCT under the parameters listed in Table 2 [2]. That is, CBCT showed higher spatial resolution than the fan beam CT system. Garayoa expains that the CBCT’s greater resolution is related to the smaller size detectors that are present in the CBCT system compared to fan beam CT [2].

**Table 4:** Image Quality Values Various image quality values for CBCT and FBCT are given for the study by Elstrøm, et al. [1].

Image Parameter		Uniformity Index (UI)	MTF (lp/cm) 50% / 10%	LCV	Mean HU in Water Equiv.
CBCT	SDH	-2.0± 0.73	5.42/9.39	1.6 ±0.1	-2.0±42.8
	SDHFS	-1.5±0.55	4.95/8.56	2.4±0.2	-3±27.4
	HQH	-2.0±0.67	5.39/9.27	3.8±0.2	-2.0±28.5
	HQHFS	-1.5±0.55	4.61/8.02	5.4±0.3	-4.0±17.1
	OBI13FS	-1.3±0.36	4.47/7.72	6.6±0.3	0±14.4
FBCT	CTMX (150 mAs)	-0.125±0.18	3.44/6.10	6.6±0.5	18±8.1
	CTMX (300 mAs)	0.125±0.12	3.39/5.99	9.6±0.3	17±5.7
	CTBB (150 mAs)	-0.25± 0.06	3.69/6.68	5.6±0.4	12±9.3
	CTBB (300 mAs)	-0.25±0.06	3.67/6.66	8.4±0.25	12±6.6

Both studies performed by Elstrøm, et al. and Garayoa, et al. generated similar results when uniformity and noise were considered [1-2]. In Elstrøm’s study, uniformity was studied by using the CTP486 module mentioned earlier [1]. When the mean values and standard deviation were studied (see Table 4) within the uniform disk, CBCT demonstrated a more accurate mean value than fan beam for the known value of '0' HU. When the standard deviation is taken into consideration, FBCT shows considerably smaller deviations compared to that of CBCT. This leads to the suggestion that fan beam CT produces images with less random noise. Scattering, beam hardening, and noise can bring about artifacts in the images. When looking at the uniformity index in Table 4 calculated by Elstrøm, et al. we can see that all CBCT scan modes produced a far greater uniformity index [1]. This was also the case in the Garayoa, et al. study where CBCT produced a much greater uniformity index and C-value as seen in Table 5 [2]. The greater UI and C-values result in images that have greater presence of artifacts in the CBCT images such as: capping, cupping, ring, and streaking artifacts. CBCT has much greater noise and artifact presence; however, the signal-to-noise ratio is lower compared to fan beam CT. The CBCT will have more grainy images, as the “true” signal will be drowned out by the large noise signal.

**Table 5:** Image Quality Values for Pelvic Protocol Various image quality values are given for CBCT and FBCT under the pelvic protocol for the study by Garayoa, et al. [2].

Scan	Filters	Signal-to-Noise Ratio	Uniformity Index	C-Value (HU)	Contrast-to-Noise Ratio
CBCT	Sharp	7.2 ± 1.1	21±3	-15±1	5.0±2.3
	Standard	10.0±1.1	21±3	-16±1	7.3±2.3
	Smooth	12.2±1.1	21±3	-15±1	11.4±2.3
Fan Beam CT	80 mAs 0.5x16	12.8±1.1	1.3±0.5	1.0±0.6	9.1±2.3
	80mAs 1x16	14.4±1.1	1.8±0.5	1.1±0.6	9.1±2.3
	80 mAs 2x16	17.8±1.1	1.3±0.5	0.4±0.6	13.6±2.3
	160 mAs 2x16	22.8±1.1	2.0±0.5	0.4±0.6	19.5±2.3
	300 mAs 2x16	30.6±1.1	1.5±0.5	0.1±0.6	23.6±2.3

Another measurement that stood out was the systems' ability to discriminate objects of low contrast. As mentioned earlier, this was referred to as the systems' low contrast visibility (LCV). From Table 4, the LCV appears to be greater in FBCT in the 300 mAs exposure tests. The lower exposure fan beam CT scans of 150 mAs also have a greater LCV than a majority of the CBCT scans [1]. These values imply that the fan beam CT scans have a greater ability to discriminate small differences in a tissue’s HU compared to the CBCT system.

Another factor that is important to consider is the dose delivered to a patient from each respective imaging modality. In the study by Kan, et al. it is clear that CBCT delivers considerably greater dose in all three of the areas scanned compared to FBCT [6]. In addition, the three scans, head and neck, chest, and pelvis, shows that CBCT produces a larger effective dose to the body and a larger absorbed dose to critical organs [6]. Each scan site can be analyzed as follows.

During the head and neck scans performed by Kan, et al., various organs were irradiated. Some critical organs to consider would be the skin, thyroid, esophagus, thymus, brain, lens of the eye, spinal cord, and others. Out of all of these organs the maximum dose from CBCT and FBCT was delivered to the thymus and secondly to the skin and lens. FBCT on the other hand irradiated the same organs but produced significantly lower doses. FBCT produces less damaging radiation dose to the critical organs and less effective dose to the body under the head and neck scan [6].

The chest scan has a longitudinal length of 13.7 cm, where the irradiated organs of interest would be the esophagus, heart, lung, and breast. The resulting scans showed that the heart received the largest dose in both CBCT and FBCT. The results show that FBCT provides significantly less absorbed dose and effective dose than its conic counterpart, CBCT under a chest scan. The pelvis scan had a longitudinal length of 13.7 cm and irradiated many vital organs such as the ovary, uterus, small intestine, bone marrow in the iliac crest, bladder, colon, rectum, and skin. The largest dose was delivered to the head and the skin for both CBCT and FBCT. This third scan tells the same story as the previous two scan sites discussed [6]. FBCT showed again that it provides a lower absorbed dose to the critical organs and lower effective dose than CBCT scans of the pelvis region. From Table 6, it can be seen that FBCT provides less absorbed dose and effective dose than the CBCT modality in every area including the thymus, heart, small intestine, skin, and overall effective body dose [6].

**Table 6:** Dose Comparison Comparison of absorbed dose to various areas and effective dose from CBCT and FBCT under a head and neck, chest, and pelvis scan [6].

Scan	Protocol	Max organ dose	Dose to skin (cGy)	Effective Dose (mSv)
FBCT	Head and Neck	Thymus 3.8 cGy	4.5	3.6
	Chest	Heart 3.0 cGy	3.0	6.9
	Pelvis	Small Intestine 3.0 cGy	3.0	10.0
CBCT	Head and Neck	Thymus 11.1 cGy	6.7	10.3
	Chest	Heart 6.7 cGy	6.4	23.6
	Pelvis	Small Intestine 6.2 cGy	5.4	22.7

### Discussion

The quantitative analysis of the image quality studies comparing cone beam CT and fan beam CT brought forth many results. The fan beam CT system appears to have lower artifact presence, less noise, greater signal-to-noise ratio, and a greater ability to discriminate low contrast objects compared to CBCT. Given that CBCT systems are intrinsically more prone to scattering, beam hardening, and artifacts, the images are grainier and less uniform. CBCT showed relative superiority in the MTF readings compared to FBCT. These greater MTF values imply that CBCT has a greater ability to distinguish small spatial variations, though its important to reiterate that these MTF values can vary greatly on protocol as mentioned earlier.

**Figure 2:**
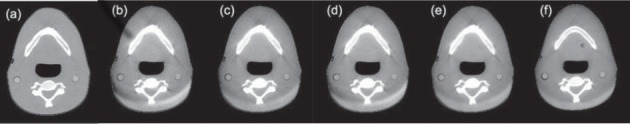
Anthropomorphic Head Phantom Reconstructed anthropomorphic head phantom images taken by (a) CT (b)-(f) OBI CBCT. From left to right on CBCT: SDH, SDHFS, HQH, HQHFS, and OBI13FS reconstructions [1].

These image quality measurements were taken to remove human bias and provide a numerical way to evaluate specific aspects of image quality. Although this is important, it may take this evaluation full-circle to apply qualitative analysis to actual images taken by the two competing systems. The images in Figure 2 were taken with an anthropomorphic head phantom by Elstrøm, et al. [1]. The CBCT images (b)-(f) show a greater presence of crescent artifact in the neck, as well as streaking throughout the image compared to the minimal artifacts with FBCT.

**Figure 3:**
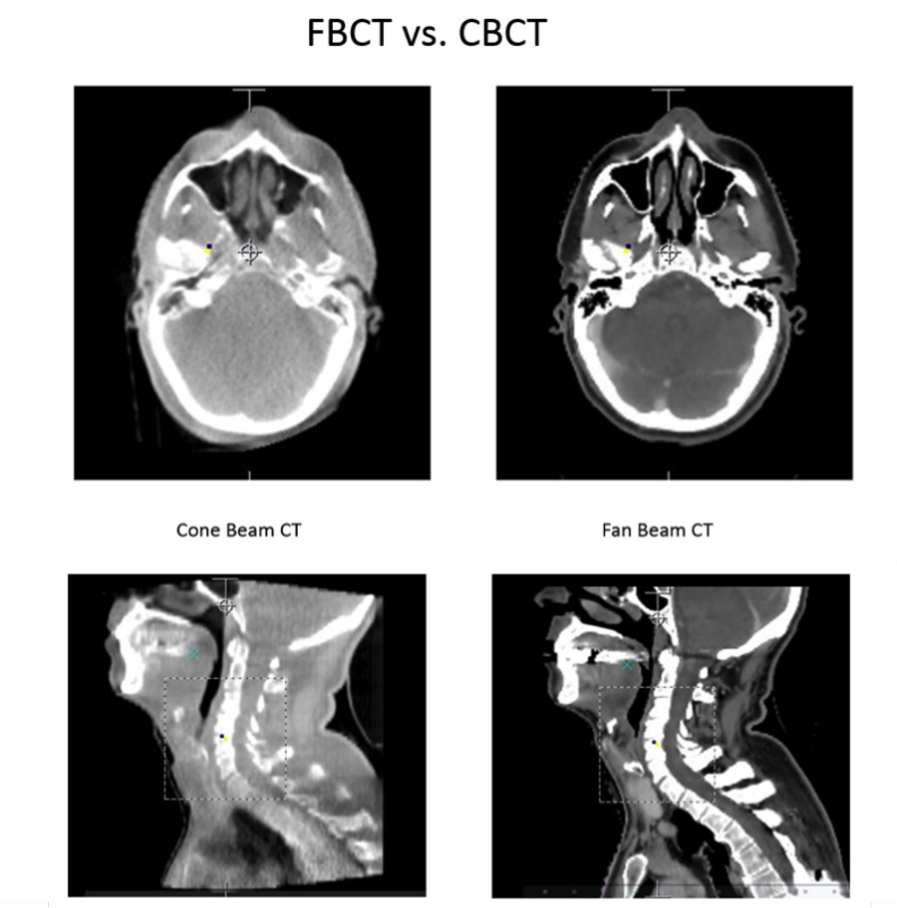
FBCT vs CBCT Cone beam CT (left) vs. fan beam CT (right) of head and neck IGRT in axial and sagittal orientation.

Figure 3 shows clinical examples of CBCT and FBCT images in axial and sagittal orientation for a head and neck IGRT case. As seen, the FBCT image is more anatomically revealing and clear. From these reconstructed images, it appears that fan beam CT systems produce better defined and more anatomically correct images compared to the cone beam CT systems.

Aside from pure image quality, the patient receives significantly less radiation per scan under FBCT than CBCT, around two to three times less dose to be more exact [6]. This aspect is important to consider, given that some treatments can have numerous fractions that require daily scans. These daily scans can cause large amounts of dose to accumulate on critical organs. Furthermore, the effective dose is considerably greater for those patients undergoing CBCT scans for IGRT, which can increase the chance of secondary cancers. In the study by Kan, et al., the linear no-threshold model describes that with a patient receiving daily treatment of 35 fractions for prostate cancer, the CBCT scans will deliver approximately 800 mSv, which could increase the secondary cancer risk by 4.0% [6].

With all of the aforementioned image parameters in mind, it can be seen from Table 7 that FBCT outperforms CBCT in various areas. Fan beam CT produces overall superior images due to the uniformity, accuracy, and clarity. These results not only indicate better image quality, it also allows the patient to receive considerably less dose with each successive scan, lowering the risk of secondary cancers and irreparable damage to critical organs. With the help of various studies discussed in this work, it can be concluded that images taken directly before treatment with CBCT suffer from a drop in quality and visualization with an increased risk of adverse health effects compared to FBCT images.

**Table 7:** Summarized Results Comparing CBCT and FBCT Summarized results from Elstrøm, et al., Garayoa, et al., and Kan, et al. showing the preferable system for various aspects [1-2, 6].

Image Criteria	Supporting Measurements	Preferrable System	
		CBCT	FBCT
Spatial Resolution	MTF	x	
Noise in Image	SNR, Water Equivalent Std Dev		x
Low Contrast Resolution	Low Constrast Visibility		x
Uniformity Image Artifacts	C- Value Uniformity Index		x
Anatomic Visualization	UI, head phantom reconstruction, C Value		x
Dose to Patient	Effective Dose, Absorbed dose to patient		x

Overall this review covers important image quality aspects regarding uniformity, noise, spatial resolution, low contrast visibility, and overall patient dose, but it may prove beneficial to extend this review to cover more aspects in the future. Further research areas should include more variety of protocols and the system’s dynamic range.
